# Integrative Diagnostic and Prognostic Paradigms in Diffuse Axonal Injury: Insights from Clinical, Histopathological, Biomolecular, Radiological, and AI-Based Perspectives

**DOI:** 10.3390/ijms26167808

**Published:** 2025-08-13

**Authors:** Alessandro Santurro, Matteo De Simone, Anis Choucha, Donato Morena, Francesca Consalvo, Daniele Romano, Pamela Terrasi, Francesco Corrivetti, Raffaele Scrofani, Nicola Narciso, Ettore Amoroso, Marco Cascella, Vittorio Fineschi, Giorgio Iaconetta

**Affiliations:** 1 Department of Medicine, Surgery and Dentistry—Schola Medica Salernitana, University of Salerno, 84081 Baronissi, Italy; asanturro@unisa.it (A.S.); fconsalvo@unisa.it (F.C.); dromano@unisa.it (D.R.); pterrasi@unisa.it (P.T.); mcascella@unisa.it (M.C.); giaconetta@unisa.it (G.I.); 2 Neuroanatomy Lab, BrainLab, Mercato San Severino, 84085 Salerno, Italy; 3 Unit of Legal Medicine, University Hospital San Giovanni di Dio e Ruggi d’Aragona, 84131 Salerno, Italy; 4 Unit of Neurosurgery, University Hospital San Giovanni di Dio e Ruggi d’Aragona, 84131 Salerno, Italy; francesco.corrivetti@sangiovannieruggi.it (F.C.); raffaele.scrofani@sangiovannieruggi.it (R.S.); nicola.narciso@sangiovannieruggi.it (N.N.); ettore.amoroso@sangiovannieruggi.it (E.A.); 5 Department of Neurosurgery, Aix Marseille University, APHM, UH Timone, 13005 Marseille, France; anis.c13@gmail.com; 6 Laboratory of Biomechanics and Application, UMRT24, Gustave Eiffel University, Aix Marseille University, 13005 Marseille, France; 7 Department of Anatomical, Histological, Forensic and Orthopedic Science, Sapienza University of Rome, 00185 Rome, Italy; donato.morena@uniroma1.it; 8 Unit of Interventional Neuroradiology, University Hospital San Giovanni di Dio e Ruggi d’Aragona, 84131 Salerno, Italy; 9 Unit of Anesthesiology, Intensive Care Medicine, and Pain Medicine, University Hospital San Giovanni di Dio e Ruggi d’Aragona, 84131 Salerno, Italy; 10 Unit of Legal Medicine, University Hospital Policlinico Umberto I, 00161 Rome, Italy

**Keywords:** DAI, TBI, neurosurgery, radiomics, AI, post-mortem, neuroanatomy, physiopathology

## Abstract

Diffuse axonal injury (DAI) is one of the most severe consequences of traumatic brain injury (TBI), characterized by widespread axonal damage in the cerebral white matter. DAI plays a crucial role in determining clinical outcomes, significantly contributing to long-term disability and mortality in severe cases. Despite advancements in neuroscience and clinical management, the diagnosis and prognosis of DAI remain challenging due to its complex pathophysiology and the difficulty of detecting axonal damage in its early stages. This study critically analyzes the clinical and post-mortem methodologies used to assess DAI, highlighting their strengths and limitations. Traditional histopathological grading systems provide valuable insights into disease progression, yet their correlation with long-term functional outcomes remains controversial. Advanced neuroimaging techniques, such as diffusion-weighted MRI, have improved lesion detection, although their routine clinical application is still limited. Additionally, emerging approaches involving biomarkers and artificial intelligence-based models hold promise for enhancing diagnostic accuracy and prognostic predictions. By synthesizing current knowledge on DAI, this work aims to outline a comprehensive framework for improving diagnosis and outcome assessment. Furthermore, it seeks to foster collaboration among clinicians and researchers, ultimately advancing the understanding of DAI and refining strategies to improve patient care.

## 1. Introduction

Diffuse axonal injury (DAI) represents a severe and often disabling consequence of traumatic brain injury (TBI), characterized by widespread axonal damage within the brain’s white matter. This condition plays a critical role in determining patient outcomes, significantly contributing to long-term disability and mortality in the most severe cases [1]. Despite advances in neuroscience and clinical care, the diagnosis and prognosis of DAI remain challenging due to its complex pathophysiology and the subtlety of its clinical presentation, particularly in the early stages. Addressing these challenges requires the integration of advanced clinical, laboratory, and imaging techniques to enable a more comprehensive understanding and management of the condition.

Significant progress has been made in elucidating the pathophysiology of DAI. Fundamental studies, such as those by Adams et al. [2], introduced a histopathological grading system that remains foundational to understanding disease progression. Nevertheless, controversies persist regarding the clinical implications of these lesion grades and their correlation with long-term outcomes [3]. Advanced imaging modalities, including diffusion-weighted MRI, have enhanced the detection of DAI; however, their potential remains underutilized in routine clinical practice. Emerging strategies, such as the use of biomarkers and artificial intelligence-based models, show promise in improving diagnostic precision and prognostic prediction [4]. Still, the absence of consensus on reliable parameters for early detection and outcome evaluation underscores the need for further research.

This perspective aims to synthesize current knowledge on DAI and identify areas for future investigation, focusing on the interplay between clinical presentation, laboratory data, and radiological findings. By critically analyzing these aspects, the study proposes a roadmap for the development of a robust diagnostic and prognostic framework. Furthermore, it seeks to promote dialogue and foster collaboration among clinicians and researchers, ultimately contributing to a deeper understanding of DAI and improving patient care strategies.

## 2. Physiopathology

A clear conceptual distinction must first be made between the two principal categories of central nervous system (CNS) injury [5].

Primary injury, occurring immediately after trauma, can be subdivided into two main forms: diffuse injury—including concussion and diffuse axonal injury (DAI)—and focal injury, such as contusions and hemorrhages resulting from direct vascular rupture [6].

Cell membrane disruption leads to the release of intracellular contents and cell death via a combination of necrotic and apoptotic processes [7].

This is followed by secondary injury, which evolves over time and is mediated by inflammatory and ischemic mechanisms. The local inflammatory response arises solely from the initial trauma, triggered by the release of antigenic molecules from damaged cells. Conversely, cerebral ischemia—either focal or global—is a secondary process, often resulting from cerebral vasospasm, particularly following intracranial hemorrhage [5].

DAI is caused by both a primary mechanical insult (axonotmesis) and a secondary progressive injury characterized by molecular axonal changes and neuroinflammatory responses [8].

The initial mechanical insult disrupts axonal integrity and axoplasmic transport, leading to the accumulation of β-amyloid precursor protein (β-APP)—a transmembrane glycoprotein abundant in the CNS [9].

Axonal damage may result from either collapse or stretching of the axolemma, leading to dysfunctional ion exchange across the membrane, neuronal depolarization, and hyperactivation of ion pumps. In particular, intracellular calcium dysregulation—triggered by mechanical membrane disruption and the excessive release of excitatory amino acids such as glutamate—plays a central role in secondary damage to axons, neurons, and vascular structures [10].

This pathophysiological cascade activates multiple intracellular signaling pathways and early transcription factors such as c-Fos and c-Jun, which regulate genes encoding for growth factors, cytoskeletal proteins, and heat shock proteins (HSPs) [8,11].

Numerous studies have documented microglial infiltration in areas with axonal disruption, underscoring the central role of glial cells in DAI pathogenesis [12,13]. Following DAI, microglia and astrocytes become rapidly activated in response to axonal disruption, releasing a spectrum of cytokines that modulate the inflammatory environment.

In fact, the pathophysiology of DAI also involves complex neuroinflammatory responses, with a prominent role played by proinflammatory cytokines and signaling pathways such as TNF-α, IL-6, and NF-κB, which further contribute to injury progression and are integral to the secondary injury cascade. The interaction between cytokine signaling and NF-κB activation constitutes a self-perpetuating loop that sustains neuroinflammation, promotes apoptosis, and impairs remyelination in DAI [14].

TNF-α contributes to secondary neuronal damage by promoting apoptosis, demyelination, and further axonal degeneration [15]. Importantly, TNF-α enhances the permeability of the blood–brain barrier (BBB), facilitating infiltration of peripheral immune cells that exacerbate neuroinflammation [16]. IL-6 elevation contributes to gliosis and chronic degenerative changes in affected neural circuits [17]. Also, the NF-κB signaling pathway is a central mediator orchestrating the transcription of inflammatory mediators post-DAI. Once activated, NF-κB translocates to the nucleus and induces the expression of cytokines such as TNF-α and IL-6, amplifying the inflammatory response [18]. Sustained NF-κB activation has been implicated in ongoing axonal degeneration and demyelination observed in DAI [19].

Recent findings also suggest dysfunction of the neurovascular–glymphatic unit in sustaining axonal damage. In particular, it has been postulated that, following traumatic injury, there can be a disruption of the cerebral vascular system as well as of the paravascular glymphatic flow. It has been demonstrated that this system is responsible for the clearance of soluble proteins, metabolic waste products (such as amyloid-beta peptides, alpha-synuclein aggregates, and hyperphosphorylated tau proteins), and excess extracellular fluid through convective flow of interstitial fluid, facilitated by the presence of aquaporins (AQP4) in the astrocytic membranes. It has been observed that, in animal models, vascular injury is associated with dilation of perivascular spaces and decreased expression of aquaporin-4 (AQP4), suggesting impaired cerebral fluid (CSF/ISF—cerebrospinal/interstitial fluid) dynamics and glymphatic clearance. This implies that traumatic insults can reduce glymphatic function by disrupting CSF-ISF exchange, thereby diminishing the brain’s capacity to eliminate interstitial waste products. Such dysfunction may perpetuate axonal and oligodendrocyte injury by hindering metabolic waste clearance and homeostatic regulation [20,21,22,23,24,25,26,27].

Oxidative stress, induced by excessive intracellular Ca^2+^, has also been implicated in the pathogenesis of DAI. This toxicity is primarily mediated within the mitochondria, where reactive oxygen species (ROS) are generated, leading to oxidative stress in the axon. The overproduction of ROS, resulting from excitotoxicity and the depletion of endogenous antioxidant defenses, triggers lipid peroxidation of cellular and vascular structures, protein oxidation, DNA damage, and inhibition of the mitochondrial electron transport chain [28,29]. These cumulative effects ultimately lead to oxidative axonal and neuronal damage.

On the other hand, recent advancements in the understanding of traumatic brain alterations underscore a paradigm shift, transitioning from viewing TBI as a singular acute event to recognizing it as a complex, chronic condition with long-term repercussions. This reconceptualization is supported by emerging evidence that highlights the multifaceted nature of TBI, encompassing a spectrum of structural, functional, and psychological alterations that may persist or even evolve over time.

Recent studies elucidate the pathophysiological processes underpinning TBI, revealing that even sub-concussive low-level impacts, yet pervasive and/or repeated, often unnoticed and untreated, can lead to significant cumulative effects and neurological impairment over time. These impacts may not result in immediate or overt symptoms but can progressively disrupt neural integrity, leading to chronic neuropsychiatric and cognitive deficits, alongside microstructural changes similar to those observed in chronic traumatic encephalopathy (CTE). The pathophysiological mechanisms underlying such processes include sustained microglial activation and chronic axonal injury, which may contribute to synaptic dysfunction and neuronal loss [30,31,32].

In this complex scenario, it is clear that determining the precise pathophysiological mechanisms of DAI can be challenging, given that different DAI phenotypes have also been identified.

### Phenotypes of Axonal Injury

Traumatic brain injury (TBI) leads to various lesions, or more accurately, to different patterns of axonal injury. This stratification occurs not only from case to case, but also within a single trauma, where different brain areas exhibit distinct forms of axonal damage [33].

Based on current evidence, five core phenotypes of axonal injury can be delineated, including sodium channel (NaCh) pathology, mechanoporation of the axolemma, amyloid precursor protein (APP) accumulation, neurofilament alterations, and calpain-mediated spectrin degradation (Table 1).

Despite this classification, there is considerable overlap between these phenotypes, making the overall complexity difficult to fully systematize.

Regarding the first phenotype, changes in sodium channel expression, distribution, and function post-DAI can contribute to neuronal excitotoxicity. Following DAI, an abnormal redistribution and upregulation of voltage-gated sodium channels (Na_v_ channels) has been described [34]. These modifications result in increased sodium influx during neuronal depolarization, disrupting ionic homeostasis. Excessive sodium entry leads to secondary calcium overload via the sodium-calcium exchanger, activating deleterious enzymatic pathways, mitochondrial dysfunction, and ultimately neurodegeneration [35]. Collectively, these findings support the existence of abnormal sodium channel activity and, in particular, sustained sodium channel-mediated excitotoxicity, which promotes apoptotic and necrotic pathways and may contribute significantly to long-term neurodegeneration [36,37].

Nodal dysfunction and alterations in the axon initial segment (AIS) further emphasize the complexity of axonal injury. Disruption of βIV-spectrin and ankyrin G in the paranodal region compromises saltatory conduction, contributing to persistent axonal dysfunction [38,39]. Importantly, nodal pathology can occur independently of APP accumulation, highlighting the necessity of a multimodal approach to the assessment of axonal injury [40].

Disruption of axonal transport, primarily identified by APP accumulation, remains the gold standard for detecting axonal injury. APP pathology is detectable as early as 30 min post-injury, peaks at 24 h, and can persist chronically, with studies reporting its presence up to one year after trauma [41]. The underlying mechanism involves microtubule destabilization, which impairs axonal transport and leads to the accumulation of cargo, resulting in axonal swellings and bulbs [42,43].

Neurofilament alteration represents another distinct phenotype, characterized by compaction, disorganization, and accumulation of neurofilaments in bulb-like or ring-shaped structures. Immunoreactivity for neurofilament light chain (NF-L) is detectable as early as 6 h post-injury [44], while more specific markers such as RMO-14—targeting epitopes exposed following disruption of neurofilament sidearms—indicate severe cytoskeletal damage [45,46]. Neurofilament abnormalities may persist for weeks to months, particularly in more severe injuries [47].

Spectrin breakdown, mediated by calcium-dependent proteases such as calpain and caspases, plays a crucial role in cytoskeletal degradation and axonal disconnection. Calpain-mediated cleavage of αII-spectrin is evident within minutes post-injury, with peak activity occurring around 6 h [48,49]. Spectrin breakdown products have been detected in both cerebrospinal fluid and serum, making them promising biomarkers for assessing TBI severity and prognosis [50,51,52].

The coexistence of these phenotypes within the same white matter tracts suggests that different axonal populations respond heterogeneously to mechanical forces. While APP accumulation and spectrin breakdown dominate early post-injury stages, sodium channel and nodal alterations tend to persist longer, implying distinct therapeutic windows for intervention [53].

## 3. Pathological Alteration and Morphological Findings

The phenotypic variability observed in axonal damage, along with its resulting heterogeneity in pathophysiological mechanisms and prognostic outcomes, underscores the complexity of DAI as a complex condition. Furthermore, the study of DAI and its diverse manifestations heavily depends on postmortem examinations, which provide invaluable insights into the morphological and molecular alterations associated with injury. These autopsy-based observations, despite inherent limitations, continue to serve as a fundamental pillar in elucidating the pathogenesis of DAI, thereby contributing significantly to the advancement of scientific knowledge in TBI research.

### 3.1. Macroscopic Findings

Currently, the diagnosis of traumatic axonal injury (TAI) is still primarily based on morphological methods. Macroscopic postmortem evaluations may reveal lesions, especially in more severe cases of DAI [54,55]. Typically, in patients who die within a few days following trauma (i.e., short-term survivors), DAI lesions are usually hemorrhagic. In those who survive for several weeks, however, gliosis may obscure the macroscopic identification of DAI.

Significantly, the macroscopic diagnosis of DAI is often subtle and ambiguous. A complementary method for observing macroscopic DAI lesions is represented by postmortem magnetic resonance imaging (PMMRI). PMMRI offers significant advantages and potentialities in the study of TBI and DAI, primarily due to its non-invasive nature and high spatial resolution, enabling detailed visualization of brain structures and pathological alterations. Additionally, PMMRI has demonstrated greater sensitivity—compared to conventional post-mortem computed tomography (PMCT) or standard autopsy inspection—in detecting subtle traumatic lesions, allowing for precise identification of hemorrhages, contusions, and DAI.

Makino et al. reported the utility of PMMRI in a TBI case with DAI [56]. The authors found that T2-weighted images revealed hyperintense areas consistent with axonal injury, while T2*-weighted sequences showed hypointense areas suggestive of hemosiderin or iron deposition in deep white matter. Moreover, although coronal section imaging of the corpus callosum showed no pathological findings macroscopically, 3D-GRE T1-weighted imaging revealed focal abnormalities indicative of DAI.

In this context, PMMRI is playing an increasingly central role in forensic neuropathology, holding substantial potential for improving diagnostic accuracy, elucidating mechanisms of injury, and correlating radiological findings with histopathology. Consequently, it is plausible that, in the future, this forensic radiology approach will be relied upon to contribute to more comprehensive medico-legal investigations of TBI.

### 3.2. Microscopic Findings

In forensic practice, the diagnosis of DAI requires microscopic confirmation through the identification of hallmark axonal pathology—specifically, swollen axonal varicosities and axonal retraction bulbs [28].

Two main criteria are used to define DAI histopathologically:

- Presence of diffuse or multifocal axonal damage in white matter tracts;
- Widespread distribution involving multiple brain regions, with at least one lesion located above and one below the tentorium [54].

The microscopic features of axonal damage vary depending on the survival time post-injury. Foundational studies by Gennarelli and Adams [2,55] defined a three-tiered grading system for DAI severity in primate models:

- Grade 1: Scattered axonal retraction balls predominantly observed in the parasagittal white matter of the cerebral hemispheres, corpus callosum, and brainstem, with occasional involvement of the cerebellum;
- Grade 2: In addition to axonal damage in the white matter of the cerebral hemisphere, a focal lesion in the corpus callosum;
- Grade 3: Widespread cerebral white matter damage combined with focal lesions in both the corpus callosum and the dorsolateral quadrant of the rostral brainstem [28].

**Figure 1:**
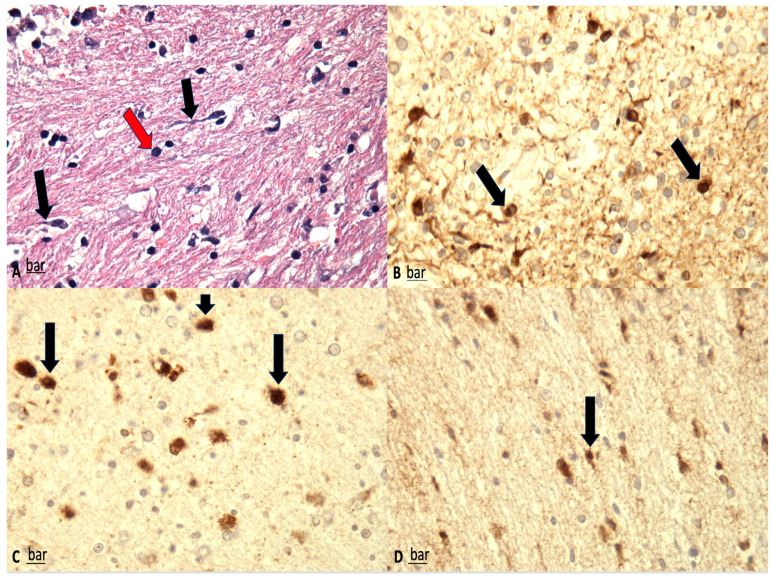
(**A**) Shrunken neurons associated with dark cell change (red arrows) and peri-neuronal vacuolation and swollen axons (black arrows) are visible (H&E, ×100). (**B**–**D**) β-APP immunostaining; significant reduction in β-APP-positive neurons, correlating well with the findings on H&E staining. Furthermore, β-APP-positive axons were fragmented and meandered, with multiple axonal retraction balls (black arrows) (magnification: ×150 (**B**), ×200 (**C**), ×100 (**D**).

Microscopically, DAI is characterized by widespread axonal disruption. A key finding is the presence of axonal retraction balls (RBs)—eosinophilic round or oval structures—most notably in the corpus callosum and dorsolateral brainstem (Figure 1). These result from mechanical shearing forces at impact, leading to axonal disconnection. Immunostaining for neurofilament proteins can reveal axonal swellings as early as 1–2 h post-injury, while silver staining becomes useful after around 8 h. In intermediate survival cases (days to weeks), microglial stars are seen, indicating a neuroinflammatory response. In long-term survivors, white matter degeneration is observed, confirming ongoing axonal pathology [57,58].

### 3.3. Immunohistochemistry

**Figure 2:**
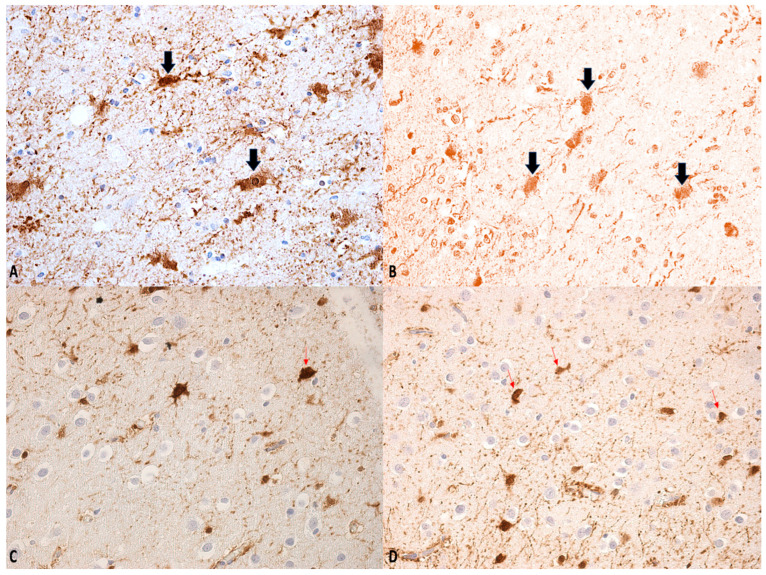
(**A**–**D**) β-APP staining: swollen axons. Regions within the bulb exhibiting a degree of immunological inactivity, suggesting that the process may be entering a phase of decline (see arrows). Magnification: ×150 (**A**–**C**); ×100 (**D**).

**Figure 3:**
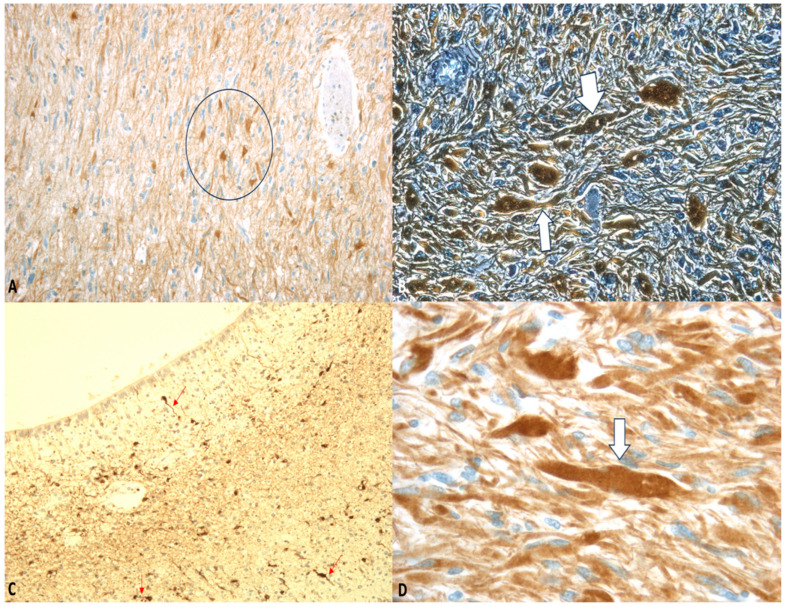
(**A**–**D**) GFAP staining: “Axonal varicosities” (within the circle). Beaded or segmented structures are indicated by the arrows. The white arrows highlight areas where the axon has been damaged and show varicosities. Magnification: ×75 (**A**), ×150 (**B**), ×75 (**C**), ×300 (**D**).

The primary immunohistochemical markers employed in the evaluation of DAI include β-amyloid precursor protein (β-APP) and glial fibrillary acid protein (GFAP) (Figure 2 and Figure 3) [8,59]. Astrocytic immunoreactivity for GFAP and S100 has proven valuable for estimating the timing of brain injury, with significantly reduced positivity observed in subacute or delayed cases. Furthermore, a potential correlation between the severity of trauma and the degree of GFAP/S100 immunopositivity has been reported [60].

Neurofilaments, as essential components of the neuronal cytoskeleton, are crucial for maintaining axonal caliber. Post-traumatic analyses have demonstrated elevated levels of light neurofilament in injured axons [61].

Spectrin, another key neuronal protein, forms periodic structures alternating with actin and adducin. The spatial arrangement of these proteins exhibits remarkable consistency, with the distance between adjacent actin-adducin rings corresponding to the length of a spectrin tetramer.

As previously discussed, oxidative stress plays a critical role in mediating both primary injury and secondary pathological progression following DAI. Among various oxidative stress biomarkers, 8-hydroxy-2′-deoxyguanosine (8-OHdG)—a product of guanine oxidation in DNA—has emerged as a particularly sensitive indicator of oxidative DNA damage. Quantitative analysis of cellular 8-OHdG levels has been established as a reliable method for assessing oxidative stress burden [62]. Recent studies support this evidence, demonstrating significantly increased 8-OHdG immunoreactivity in post-mortem brain tissue from DAI fatalities compared to controls [8].

For the neuropathological diagnosis of DAI, the visualization of β-APP accumulation in damaged axons has been considered the gold standard. Interestingly, despite all axons within a specific white matter tract being exposed to similar high strains and strain rates during DAI, only a portion exhibit β-APP accumulation. This suggests that β-APP, often considered a marker of axonal injury, may not detect all affected axons at a specific point after DAI. Consistent with this, earlier research in rodent models demonstrated that compacted neurofilament medium (RMO-14) can identify injured axons that do not show β-APP accumulation [63]. Additionally, subsequent studies revealed that a proteolytic fragment of spectrin alpha-II, known as “SNTF,” can detect a subpopulation of degenerating axons that remain undetected by the standard transport disruption marker, β-APP. Johnson et al. reported increased axonal co-localization of SNTF with β-APP following severe DAI, with a subset of SNTF-positive axons lacking β-APP accumulation. Notably, some co-localization was observed between SNTF and less abundant neurofilament subtype markers. Other SNTF-positive axons, however, did not co-localize with any other marker. Similarly, axonal pathology positive for RMO-14 existed independently of SNTF and β-APP [45,64]. These immunohistochemistry studies highlight the heterogeneity of DAI phenotypes.

## 4. Clinical Severity Grading

### Timing for Surgery

The Brain Trauma Foundation’s Guidelines for the Management of Severe Traumatic Brain Injury [65], following the results of the RESCUEicp study and data from the DECRA (Decompressive Craniectomy in Patients with Severe Traumatic Brain Injury) study [66,67], have led to the generation of three new recommendations. These guidelines remain the most important scientific authority in the management and prevention of secondary injuries, effectively dictating the timing for surgery.

However, the published guidelines and studies, beyond establishing evidence that wide craniotomy is superior to mini craniotomy in terms of efficacy and benefit on intracranial pressure (ICP), leave ample room for debate. For instance, is bifrontal craniotomy better than lateral craniotomy? Additionally, it is not well established when to prefer one over the other. Moreover, the impact of craniotomy timing on diffuse axonal injury (DAI) is not covered in the guidelines. All these issues represent important perspectives for further investigation. In this regard, an interesting piece of evidence comes from a guinea pig study, which aimed to evaluate the hypothesis that reduced increases in ICP after experimental TBI are necessary for decreased axonal damage and white matter atrophy in mice [68]. Study results indicated that decompressive craniectomy was associated with reductions in ICP and a decrease in peri-contusive axonal damage and white matter atrophy. Additionally, previous tests in rats had demonstrated that when dividing rats into two groups based on the timing of the ICP elevation decrease following injury, the group with sustained ICP elevation showed greater neuronal damage. Conventional histological evaluations revealed greater neuronal damage, potentially associated with the redistribution of cathepsin-B from the lysosomal compartment to the cytosol, giving a signal of cell death [69]. These results suggest that a persistent increase in ICP exacerbates plasmalemmal disruption of neurons, which could lead to persistent neuronal impairment and eventual neuronal death [70]. However, it must be considered that significant translational limitations exist that pose challenges in accurately replicating the complex pathology of human DAI and in extrapolating preclinical findings to clinical settings.

Especially in moderate trauma, where the indication for craniectomy is more nuanced because the clinical parameters are themselves more nuanced, recognizing an increase in ICP is critical. An important piece of evidence comes from a study by Hamdeh et al. In a cohort of 52 patients with TBI, they found that an increase in ICP occurs in about one-third of patients with severe TBI presenting with DAI, finding that age and lesions on DWI sequences in the midbrain were associated with increased ICP. These results suggest that the localization of lesions on MRI may help predict increased ICP in patients with DAI [71].

## 5. Outcome Assessment

### 5.1. Clinical Prognostic Markers

Neurological examination remains essential in detecting clinical deterioration in patients under neurocritical care following TBI. The traditional neurological assessment involves evaluating pupillary reflexes, motor function, verbal responses, and consciousness level, typically assessed through a grading scale. Since new-onset, unresponsive anisocoria often occurs in these situations, it is crucial to evaluate early life-threatening elevations in ICP, which is recognized as an independent factor in mortality and morbidity [72]. Consequently, ICP assessment remains a pivotal element in prognostic determination following trauma. Invasive monitoring methods, such as intraparenchymal or intraventricular probes, can be employed; however, these techniques are subject to limited reproducibility as they depend on factors like instrument calibration, operator experience, and proper probe placement. Thus, their routine use is not recommended.

Several non-invasive assessment techniques are available, primarily focusing on pupillary evaluation. Specifically, quantitative pupillometry measures various parameters, including pupil constriction velocity (CV) in response to light (in mm/s), dilation velocity (DV) in the absence of light (in mm/s) and pupil size (PS) (in mm). This approach offers a degree of reproducibility and, when performed repeatedly, appears to be a promising tool for non-invasive ICP monitoring in patients with TBI [73].

It is important to note that DAI does not often correlate with increases in ICP, making identification strategies based solely on pupillary assessment challenging. A study involving a cohort of 52 patients demonstrated that increased ICP occurs in only a subset of individuals, comprising roughly one-third of individuals with severe TBI exhibiting DAI [74].

Another key clinical prognostic tool is the Glasgow Coma Scale (GCS), a universally recognized scale widely used in all emergency settings to evaluate the neurological status following trauma at the first medical contact. Since its introduction by Teasdale and Jennett in 1974 [75], the GCS has been embraced globally for its simplicity, reproducibility, and rapid application across various clinical settings. The GCS score, obtained by summing the scores of the three subscales (ocular response, verbal response, and motor response), can range from 3 to 15; scores indicate more severe impairment and, likely, poorer prognosis. In patients with DAI, a lower GCS score on admission correlates with higher mortality and with greater dependency post-injury [76,77].

Furthermore, as reported by Skandsen et al. [78], GCS correlates with clinical outcome predominantly when DAI is markedly present (r = 0.47; *p* = 0.001), emphasizing the importance of accurately diagnosing this lesion type. In their cohort of 159 patients, the presence of lesions occurring in the dorsolateral brainstem was identified as the main determinant of morbidity at follow-up. However, other studies suggest that prognosis correlates more strongly with lesions of the corpus callosum rather than the brainstem [79].

In another study by Chelly et al., involving a cohort of 124 patients, multivariate analysis identified several factors associated with increased mortality, including dysautonomia (*p* = 0.018; odds ratio [OR] = 4.17), hyperglycemia ≥ 8 mmol/L (*p* = 0.001; OR = 3.84) at the time of ICU admission, and subdural hematoma (*p* = 0.031; OR = 3. 99). Factors associated with an unfavorable outcome on the Glasgow Outcome Scale included GCS score < 8 (*p* = 0.032, OR = 3.55), secondary systemic injury score ≥ 3 (*p* = 0.034, OR = 2.83), hyperglycemia ≥ 8 mmol/L (*p* = 0.002, OR = 5.55), and the presence of ≥6 DAI lesions (*p* = 0.035, OR = 3.33). In patients with pure DAI, absence of recovery of consciousness was the only independent factor of mortality (*p* < 0.001, OR = 116.4), while only the need for transfusion was an independent factor of adverse outcome (*p* = 0.017, OR = 4.44) [80].

Additionally, Mata-Mbemba et al. [81] conducted a study on 140 patients with TBI in Japan, demonstrating that intraventricular hemorrhage was an independent predictor of DAI (OR 4.2, 95% CI = 1.3–14.3).

These findings highlight the importance of establishing a comprehensive framework for interpreting individual clinical, laboratory, and radiological data to better understand and predict outcomes in DAI cases. While clinical examination remains indispensable for diagnosing DAI, further research is needed to identify parameters most strongly associated with its presence, ideally confirmed through laboratory tests and neuroimaging.

### 5.2. Serum Biomarkers

The role of serum biomarkers in the diagnosis and prognosis of DAI has been extensively investigated through a multidisciplinary approach encompassing both laboratory-based experiments and clinical research studies. These investigations have identified a variety of molecular and cellular markers that reflect underlying pathophysiological processes such as axonal degeneration, neuronal injury, and secondary neuroinflammatory responses.

#### 5.2.1. Neural Markers

Neuron-specific Enolase (NSE) is among the most extensively studied markers of neural injury. This isoenzyme of enolase is localized in the neuronal cytoplasm and is a reliable indicator of DAI [82]. Although serum NSE levels have been shown to increase following various types of TBI, its correlation with contusion volume is limited [83]. Moreover, due to its short half-life, serum NSE levels exhibit a strong association with clinical outcomes primarily during the acute phase of injury; subsequently, this correlation diminishes owing to several confounding factors—including sepsis, hypoperfusion, bleeding, and liver or kidney dysfunction—that can also elevate serum NSE concentrations. Therefore, some authors have proposed that the NSE level to admission/GCS score ratio (NGR) could be an effective indicator for early identification of DAI within the first hours post-injury [84].

Ubiquitin C-terminal hydrolases L1 (UCH-L1) represents a more specific neuronal marker. UCH-L1 is part of a family of deubiquitinating enzymes, capable of removing ubiquitin from their protein substrates. Recent trials have demonstrated consistent results regarding UCH-L1, which shows a strong association with central nervous system injury, predominantly detectable in neuronal cell bodies [85]. Studies have revealed significant correlations between cerebrospinal fluid (CSF) and serum UCH-L1 levels in severe TBI patients, with relationships observed to clinical outcomes and 3-month mortality [86]. However, no studies to date have specifically investigated the role of UCH-L1 in DAI.

Tau protein, a microtubule-associated protein and a fundamental component of the axonal cytoskeleton, has also been investigated. Although the pathological aggregation of tau has been extensively studied in Alzheimer’s disease (AD), several authors have reported an increase in acetylated tau following brain injury. This marker accumulates in the blood during the acute phase of brain injury, contributing to axonal degeneration and neurological impairment [87]. In a study involving 288 professional hockey players, tau levels increased significantly after concussion (4.5 pg/mL [0.06 to 22.7] vs. 10.0 pg/mL [2.0 to 171], *p* < 0.001) and demonstrated higher diagnostic accuracy compared with other established brain injury biomarkers such as S-100B and NSE (AUC: tau = 0.80, S100 = 0.67, NSE = 0.55) [88].

Other studies have confirmed the critical role of tau protein in outcome prediction, with higher tau levels associated with unfavorable prognosis [89]. Additional research evaluated tau concentrations in DAI patients, demonstrating significantly elevated levels within 6 h of injury compared to non-DAI controls [90].

#### 5.2.2. Glial Markers

S100-B is one of the most active members of the S100 protein family; it performs a wide range of functions, including calcium-binding and regulation of ribonucleic acid synthesis [91]. This protein is predominantly secreted by astrocytes and other glial cells; however, some studies have demonstrated that it can also be produced by adipocytes. While multiple studies suggest that S100-B is released following brain insults and its serum levels correlate directly with the degree of injury and outcome [92], other research has re-evaluated its role in severe brain trauma, indicating that its serum levels are limited by the integrity of the blood–brain barrier (BBB). Additionally, its short half-life results in a narrow sampling window, and its extracerebral sources of production further complicate its use as a biomarker, making it less ideal for evaluating DAI [93].

Glial fibrillary acidic protein (GFAP) is an intermediate filament protein expressed almost exclusively in astrocytes’ cytoskeleton. Consequently, GFAP is a more specific marker of glial injury and BBB disruption. Several studies have confirmed its utility as not only a discrimination marker but also as a predictor of poor outcomes in severe TBI [94,95].

#### 5.2.3. Inflammatory Markers and Miscellanea

Tumor Necrosis Factor-α (TNF-α) is a proinflammatory cytokine that initiates the inflammatory cascade and is activated by a variety of stimuli, including trauma, infection, and brain injury. It is primarily produced by microglia and astrocytes within the central nervous system (CNS), and its concentration increases in serum and CSF during the first hours following TBI. However, studies have not demonstrated a significant association between serum TNF-α levels and clinical outcomes in TBI and DAI, likely because TNF-α serum levels are influenced by a combination of polytrauma and brain injury [96].

Interleukin-6 (IL-6) is a well-studied proinflammatory cytokine released in response to acute stressors, including CNS injury. Elevated IL-6 levels are observed during CNS inflammatory states, leading some authors to suggest a possible contribution to secondary neurological injury in TBI [97]. Although several studies have reported a correlation between severe TBI and poor outcomes [98,99], data regarding IL-6 levels are conflicting concerning its utility as an indicator of increased ICP following TBI [100,101].

Lipoprotein (i.e., high-density lipoprotein HDL) plays a vital role in the transport of lipids and cholesterol in human plasma and can be present in the CNS due to its capacity to cross the BBB [102]. Zhong et al. found elevated HDL levels during the first week post-injury in a cohort of 70 DAI patients compared to 106 patients with non-DAI TBI. They identified HDL as an independent predictor of DAI [103].

Finally, recent studies have investigated the potential modulatory role of progesterone in influencing outcomes in DAI patients. Evidence suggests that progesterone therapy may improve the outcome in DAI patients, likely through modulation of cytokine levels and reduction in injury and oxidative stress activity [104].

### 5.3. Radiological Assessment of Diffuse Axonal Injury (DAI) in Adult and Pediatric Patients

**Figure 4:**
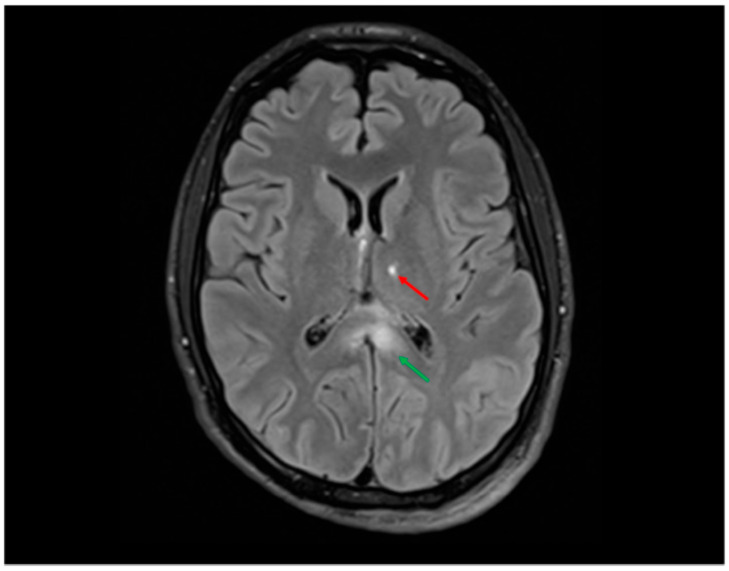
FLAIR sequence showing hyperintensities in the splenium of the corpus callosum (≈12 mm, green arrow) and at the left thalamo-capsular junction (≈9 mm, red arrow) in a young man involved in a high-speed motor vehicle collision (MVC). These locations are typical of grade II DAI according to the Adams classification and are frequently associated with impaired consciousness, cognitive deficits, and motor dysfunction. In this case, the patient presented with prolonged coma in the acute phase, in line with the neuroradiological findings.

DAI predominantly affects the subcortical and deep white matter, particularly the corpus callosum, fornix, and internal capsule, with less involvement of the midbrain and pons. Initial non-contrast computed tomography (CT) may reveal mild swelling but often fails to detect the extent of axonal damage. Magnetic resonance imaging (MRI) is preferred for assessment: since most DAI lesions are non-hemorrhagic, T1-weighted MRI may appear normal in the early stages. T2-weighted imaging (T2WI) and fluid-attenuated inversion recovery (FLAIR) sequences can demonstrate hyperintense foci in the subcortical white matter and corpus callosum, with multifocal involvement being typical (Figure 4). Lesions are often microscopic and scattered, contributing to the discrepancy between clinical presentation and imaging findings in DAI. Hemorrhagic components appear as hypointense signals on T2*-weighted imaging and susceptibility-weighted imaging (SWI), with SWI being more sensitive than gradient-echo (GRE) sequences in detecting microbleeds. Lesion burden and distribution frequently correlate with injury severity and long-term neurological outcomes [105].

#### 5.3.1. Radiological Findings in Adult DAI

In adults, imaging biomarkers are increasingly used alongside continuous intracranial pressure (ICP) monitoring to assist in prognosis and clinical decision-making. Several studies have explored the predictive value of CT findings for ICP levels and long-term outcomes. For example, sulcal effacement and third ventricular compression observed on initial CT scans are strong indicators of elevated ICP, with these features correlating with maximum and mean ICP values. These findings suggest that combining radiological markers with direct ICP measurements can improve early prognostication and patient management [71].

Although ICP elevation is less common in DAI compared to focal TBI, it remains a critical factor in secondary brain injury. A study on DAI indicated that approximately one-third of patients with severe TBI and DAI exhibit elevated ICP, underscoring the importance of close monitoring. Notably, lesions in the substantia nigra and tegmentum (SN-T) were of particular interest; non-hemorrhagic lesions in these regions, visible on diffusion-weighted imaging (DWI), were associated with prolonged periods of elevated ICP, suggesting a more severe injury mechanism. Furthermore, younger age and the presence of non-hemorrhagic SN-T lesions on DWI were identified as significant predictors of ICP levels above 20 mm Hg, highlighting the value of early MRI in guiding clinical management [106].

The study by Ravikanth et al. emphasizes the important role of MRI in assessing DAI severity, alongside GCS motor score and hemorrhagic lesions, in predicting recovery and length of hospital stay. Their findings showed that patients with small hemorrhagic lesions in the lobar white matter typically regained consciousness within 1–2 weeks, whereas those with additional lesions in the corpus callosum required 3–4 weeks. Patients with brainstem lesions needed more than 3–4 months for recovery. Hospital stay durations also varied: Grade I DAI required 2–3 weeks, Grade II 3–4 weeks, and Grade III 7–8 weeks. Hemorrhagic lesions and traumatic space-occupying injuries were associated with poorer outcomes. The presence of brainstem injuries and low GCS motor scores at 24 h post-admission further correlated with unfavorable prognoses [107].

**Figure 5:**
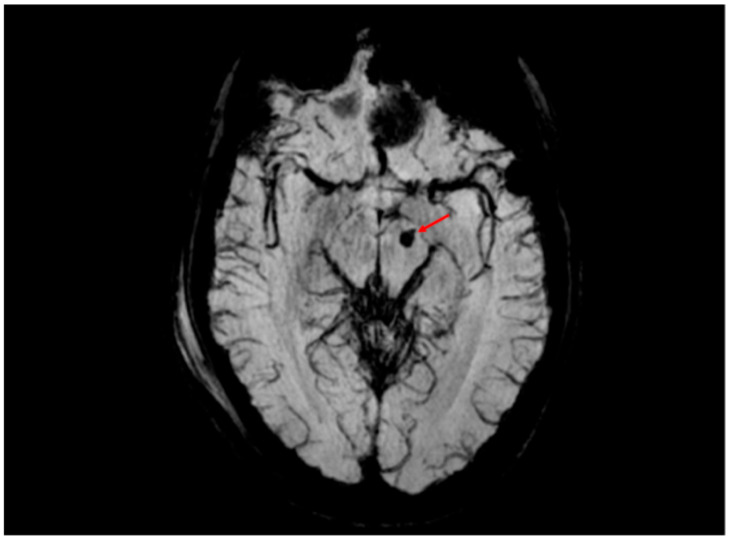
SWI sequence revealing a 4 mm hemorrhagic lesion (volume of approximately 0.03 cm^3^, red arrow) in the left cerebral peduncle, consistent with hemorrhagic diffuse axonal injury (DAI). This finding corresponds to grade III DAI according to the Adams classification, which includes hemorrhagic lesions involving deep brain structures such as the brainstem. The anatomical location within the left cerebral peduncle suggests involvement of the corticospinal tract and correlates with contralateral motor deficits observed in the clinical examination of the patient. SWI provides superior sensitivity in detecting cerebral microhemorrhages, which are critical for prognostic stratification.

**Figure 6:**
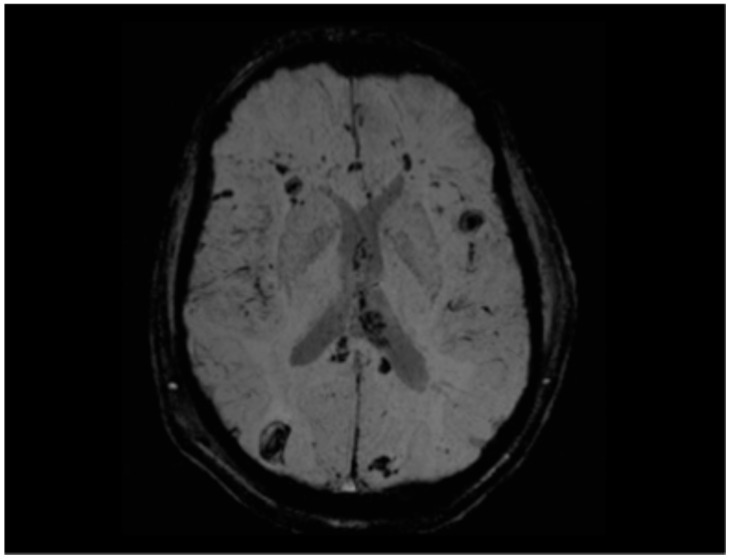
SWI sequence showing multiple foci of hemorrhagic diffuse axonal injury in the bilateral front-to-parieto-temporo-occipital subcortical white matter, right occipital region, and left external capsule, in a young man involved in a high-speed motor vehicle collision (MVC). Involvement of the corpus callosum is also evident, with a particularly large hemorrhagic focus in the splenium. Several lesions—particularly in the right occipital lobe, corpus callosum, and left external capsule—exhibit a larger ovoid morphology, in line with the variable appearance reported in diffuse axonal injury. Manual estimation identified over 20 foci, ranging from 2 mm to >10 mm in diameter, with an estimated cumulative lesion volume of approximately 1.5–2.0 cm^3^. This quantitative and morphologic assessment underscores the severity and extent of axonal injury and contributes to prognostic stratification. This pattern corresponds to grade III diffuse axonal injury according to the Adams classification. The distribution of the lesions is anatomically consistent with the patient’s visuospatial dysfunction and contralateral motor deficits.

Hamdeh et al. investigated the use of MRI within the first week post-trauma in patients with severe TBI. They observed that many patients exhibited lesions in the brainstem, hemispheres, and corpus callosum. Interestingly, grading of DAI based on Adams’ classification did not correlate with clinical outcomes. However, the presence of brainstem lesions—in particular, those involving the substantia nigra and tegmentum, as identified by SWI—served as a strong independent predictor of poor long-term outcomes (Figure 5 and Figure 6). The study proposes an extended anatomical MRI classification system for DAI aimed at improving early outcome prediction [71].

#### 5.3.2. Radiological Findings in Pediatric DAI

In pediatric patients, early MRI biomarkers have demonstrated a crucial role in predicting long-term outcomes following severe TBI. A study involving 233 children found that specific MRI features—such as contusion volume, ischemic regions, and brainstem injury—independently predicted long-term outcomes beyond the core IMPACT clinical variables. Notably, brainstem lesions, often manifesting as microhemorrhages linked to DAI, were associated with worse outcomes, highlighting the significance of lesion depth rather than overall DAI burden for prognosis. The inclusion of these early MRI markers significantly enhanced outcome prediction models at 6- and 12-month follow-ups [108].

In another pediatric study, Janas et al. evaluated whether early MRI findings could improve the predictive accuracy of the IMPACT model. While higher DAI grades on early MRI correlated with poorer 6-month functional outcomes, incorporating DAI grade into the IMPACT model did not significantly improve the prediction of adverse neurological outcomes. This suggests that further research is necessary to clarify the precise role of early MRI in outcome prognostication [109].

Additional studies indicated that the depth of diffusion restriction lesions correlated with delayed recovery of command-following abilities. Similarly, deeper FLAIR lesions in the brainstem were associated with worse outcomes as measured by the GOSE-Peds score [110,111,112]. Baker et al. also found that FLAIR lesions and microhemorrhages within the brainstem correlated with poorer outcomes in children who underwent craniectomy, emphasizing the importance of early neuroimaging in prognostic assessment [113].

Recent studies have highlighted the promising role of advanced MRI techniques, such as diffusion tensor imaging (DTI), in detecting subtle axonal injuries and providing critical insights into the prognosis of pediatric TBI patients. DTI measures several key parameters, including Fractional Anisotropy (FA), Mean Diffusivity (MD), and Axial Diffusivity (AD), which reflect white matter integrity. FA indicates the directional preference of water diffusion, with decreased values suggestive of axonal damage. MD measures overall water diffusion, with elevated values indicating tissue damage or edema. AD specifically assesses diffusion along the principal axis of the axon, offering additional assessment of axonal integrity. These parameters are crucial in detecting microscopic white matter injuries not visible with conventional imaging. Despite its advantages, DTI has limitations such as sensitivity to patient motion, difficulty distinguishing crossing fibers within a voxel, and variability due to differing acquisition protocols across studies.

Overall, these investigations underscore the significant prognostic value of early MRI in both pediatric and adult DAI cases. Early detection and characterization of lesions can refine clinical decision-making, guide management, and improve outcome predictions—particularly pertinent in pediatric populations within clinical trials. Nonetheless, the technical complexities of DTI and data comparison across studies pose challenges for routine clinical application. Despite this, DTI remains a valuable tool for prognosis, monitoring recovery, and individualizing treatment strategies in TBI patients [114].

In recent years, several studies have explored the prognostic value of traumatic axonal injury (TAI) in TBI, using different MRI techniques. One of the most recent and innovative studies in this field explores the location, number, and volume of TAI lesions in TBI patients, with a focus on their prognostic value. Notably, the “Trondheim TAI-MRI grading” system was developed to classify TAI based on early MRI findings. In this grading system,

- Grade 1 refers to TAI located in the hemispheres or cerebellum, representing the least severe injury category;
- Grade 2 corresponds to TAI located in the corpus callosum;
- Grade 3 encompasses unilateral TAI in the thalamus or brainstem and bilateral TAI in the basal ganglia;
- Grade 4 includes bilateral TAI in the mesencephalon or thalami, also correlating with worse prognosis;
- Grade 5 denotes bilateral TAI in the pons, identified as the most severe and strongly predictive of poor outcomes.

This study indicated that TAI lesion volume and contusions seen on FLAIR sequences were key outcome predictors, especially in severe TBI. Quantitative models based on FLAIR volumes outperformed traditional clinical grading systems, providing more accurate prognoses by accounting for total lesion burden. This emphasizes the value of quantitative imaging in improving outcome predictions in TBI patients. The authors also proposed that integrating artificial intelligence (AI) into this grading scheme could further augment its clinical utility, enabling more precise and automated prognosis and facilitating personalized patient management [115].

As the field evolves, future research should focus on refining these advanced imaging techniques and incorporating AI algorithms to automate lesion detection and outcome prediction. Such integration could enhance clinical workflows and optimize treatment strategies [116,117]. Longitudinal studies with serial early MRI scans—particularly in pediatric populations—will be essential for improving early diagnosis, monitoring evolution, and managing long-term DAI. Additionally, identifying MRI biomarkers associated with ICP elevation, especially in brainstem injuries, could provide valuable insights for timely interventions and personalized treatment approaches. By combining cutting-edge imaging modalities with AI-driven analysis, it is possible to move toward a more accurate and individualized management of DAI, ultimately improving outcomes for patients with TBI.

### 5.4. DAI and Artificial Intelligence

Rooted in computational sciences and powered by robust databases, AI and its subfields machine learning (ML) and deep learning (DL) are implemented to autonomously identify patterns in data, learning and problem-solving tasks. This technology has gained significant interest in TBI with AI-driven algorithms being developed to enhance imaging interpretation, prognosis prediction, and critical care management. However, few AI models focus exclusively on DAI. The earliest AI-based model sought to improve DAI diagnosis in patients with mild TBI [118]. More recently, emerging approaches include transformer-based models, self-supervised learning, and federated learning in the analysis of neuroimaging data relevant to TBI and DAI [119,120,121].

#### 5.4.1. Machine Learning-Based Prognosis Models

Tjerkaski et al. [122] developed a ML model to enhance prognosis prediction in TBI with DAI. They retrospectively analyzed MRI scans from 351 critically ill patients using FLAIR, DWI, and SWI, all of which can detect DAI with varying sensitivity. A genetic ML algorithm identified combinations of area of the brain affected by DAI, using each different MRI modality. These multiple combinations were then tested by the algorithm, searching for correlation with long term clinical outcome (defined by the GCS). Then, these features were combined with clinical predictors, such as age, GCS, and pupillary response, and processed through a random forest algorithm, improving the model’s performance (AUC from 0.67 to 0.72). While this model did not achieve outstanding predictive accuracy, it provided valuable insights, particularly by challenging traditional grading systems, such as that of Adams et al. [2], which treat all brainstem DAI equally. Tjerkaski et al. demonstrated the nuanced impact of DAI severity across different brain regions, underscoring the need for refined imaging-based grading approaches in TBI prognosis. This study identified the midbrain tegmentum, splenium of the corpus callosum, and posterior limb of the internal capsule as critical regions influencing prognosis. Notably, midbrain tegmentum lesions were consistently linked to unfavorable outcomes, regardless of the MRI sequence. These insights formed the basis of the Stockholm MRI grading system, which challenged traditional approaches and is currently being studied through a prospective multicentric study [123]. A recent advancement in this domain is represented by the development of the Automated Surgical Intervention Support Tool for TBI (ASIST-TBI), a deep learning model designed to predict the need for neurosurgical intervention directly from acute CT scans. Trained on a large retrospective dataset and validated on a consecutive prospective cohort, ASIST-TBI demonstrated high diagnostic accuracy (AUC up to 0.92), maintaining robust performance in real-world testing. These findings highlight the growing potential of AI-driven tools to assist clinical decision-making and triage in acute neurotrauma settings [124,125].

#### 5.4.2. Improved Diagnosis and Prognosis Through Deep Learning

Danilov et al.’s [126] study stands out for its dedicated focus on DAI, aiming to improve diagnosis and prognosis through advanced imaging techniques and radiomics. Radiomics involves extracting quantitative imaging features using DL algorithms, such as convolutional neural networks (CNN). These features are then evaluated by algorithms to determine whether they are associated with a specific diagnosis or clinical outcome. In this study, radiomics were applied to diffusion kurtosis imaging (DKI), an advanced MRI modality that extends diffusion tensor imaging (DTI). Unlike DTI, which measures overall water diffusion, DKI evaluates the kurtosis (or skewness) of water diffusion, providing a more detailed analysis of microstructural changes, including areas of restricted diffusion that may signal neuronal damage. The authors analyzed 342,300 radiomics features derived from DKI scans of 31 DAI patients and 12 healthy controls. Among these radiomics, nine features emerged as significant biomarkers (*p* < 0.0001) with robust diagnostic and prognostic capabilities. These markers could distinguish healthy individuals from DAI patients and further stratify the latter into groups with favorable or unfavorable outcomes. Favorable outcomes were defined as achieving a Functional Independence Measure (FIM) score of ≥100 at three months post-injury. The model demonstrated high accuracy, exceeding 0.9, in differentiating brain states and predicting patient outcomes. While these findings highlight the potential of DKI-based radiomics to outperform traditional region-of-interest (ROI) analyses, their results are based on a small cohort of 43 individuals and require external validation. Mohamed et al. [106] also used DL to develop a model to predict the outcome of patients with DAI, focusing on brainstem lesions. Through a total of 38 patients, they generated 725 MRI sections in which a CNN model interpreted the brain stem injury to generate outcome predictions. The model was able to predict GCS outcomes with a specificity of 0.43 and a sensitivity of 0.997. It showed an AUC of 0.917.

## 6. Conclusions

Research on diffuse axonal injury (DAI) remains a critical priority, as it continues to represent a major cause of mortality and long-term neurological disability worldwide.

This perspective acknowledges that axonal and glial damage, neuroinflammation, oxidative stress, and disruption of neurovascular–glymphatic homeostasis may evolve over time, often persisting well beyond the initial traumatic event. These delayed and progressive processes call for long-term surveillance and a multidimensional diagnostic framework that integrates histopathological, radiological, and biomolecular data.

Over the past decade, a growing body of evidence has reshaped our understanding of traumatic brain injury (TBI), prompting a shift from viewing it as a single acute insult to recognizing it as a dynamic and potentially chronic condition.

Further progress has been achieved in defining DAI phenotypes through innovative imaging modalities, including advanced MRI-based radiomics, which exhibit superior capabilities in early detection, lesion characterization, and outcome prediction, particularly when combined with emerging artificial intelligence (AI) algorithms.

For pathologists, established immunohistochemical markers such as β-APP and GFAP continue to play a crucial role in diagnostic evaluation. Nonetheless, the possible application of inflammatory markers—including immunohistochemical indicators of TBI—presents challenges in early stages due to overlapping secondary changes such as hypoxia and edema that frequently follow traumatic events.

Despite these substantial research efforts, the clinical prognosis for severe (high-grade) DAI remains generally unfavorable [8]. Challenges continue to include standardizing diagnostic criteria, translating laboratory findings into clinical protocols, and refining individualized treatment strategies. Moving forward, understanding the molecular mechanisms underlying TBI pathogenesis—particularly intracellular ROS homeostasis, oxidative stress, Ca^2+^-mediated cellular metabolism, and apoptotic pathways—remains essential.

Future research should focus on refining multimodal diagnostic frameworks, validating AI-driven prognostic models, and conducting longitudinal studies in both pediatric and adult populations. In this context, the necessary integration of histopathological, radiological, and biomolecular data will be essential to comprehensively understand the heterogeneity and progression of DAI [127,128,129,130,131,132,133,134,135]. Ultimately, the integration of these diagnostic and prognostic paradigms could play a pivotal role in developing personalized therapeutic strategies, enhancing prognostic accuracy, and improving patient management and long-term outcomes in DAI.

## Figures and Tables

**Table 1:** Comparative overview of the five main phenotypes of axonal injury observed in diffuse axonal injury (DAI).

Phenotype	Pathological Feature	Clinical Relevance	Imaging and Laboratory Correlates	Temporal Progression
Sodium Channel Pathology	Loss of Nav1.6 channels; nodal elongation; disorganization of the node of Ranvier	Disruption of action potential conduction; contributes to long-term network dysfunction	Not directly visualized; indirect evidence through advanced MRI (e.g., DTI abnormalities)	Early and persistent; may evolve independently of APP accumulation
Axolemma Mechanoporation	Physical disruption of axolemma; ionic imbalance and depolarization	Initiates cascade of ionic dysregulation (especially Ca^2+^); triggers apoptosis and secondary axonal damage	May contribute to diffuse signal abnormalities on DWI or T2WI	Immediate post-injury event; resolves or progresses based on severity
APP Accumulation	Blocked axonal transport leading to β-amyloid precursor protein build-up	Gold standard for diagnosis; indicates disrupted axonal transport	Correlates with DWI hyperintensities and microbleeds (SWI in hemorrhagic lesions)	Detectable as early as 30 min; peaks at 24 h; may persist for months
Neurofilament Alteration	Cytoskeletal damage with neurofilament compaction and accumulation	Marker of cytoskeletal disorganization; associated with structural instability of axons	Correlated with reduced FA on DTI; elevated NF-L levels in serum/CSF	Begins within hours; may persist for weeks to months
Calpain-Mediated Spectrin Degradation	Proteolytic cleavage of αII-spectrin by calpain/caspases	Indicates axonal disconnection and cytoskeletal collapse; relevant for prognosis and biomarker studies	Not visualized on imaging; detectable via serum/CSF spectrin breakdown products (SBDPs)	Rapid onset (minutes); peaks around 6 h; declines over 24–48 h

DAI, Diffuse Axonal Injury; APP, Amyloid Precursor Protein; Nav1.6, Voltage-gated Sodium Channel Type 1.6; DTI, Diffusion Tensor Imaging; DWI, Diffusion-Weighted Imaging; T2WI, T2-Weighted Imaging; SWI, Susceptibility-Weighted Imaging; FA, Fractional Anisotropy; NF-L, Neurofilament Light Chain; CSF, Cerebrospinal Fluid; SBDPs, Spectrin Breakdown Products.

## Data Availability

The data presented in this study are available on request from the corresponding authors.
